# Contraceptive prevalence rate and associated factors among reproductive age women in four emerging regions of Ethiopia: a mixed method study

**DOI:** 10.1186/s40834-021-00162-9

**Published:** 2021-06-01

**Authors:** Delayehu Bekele, Feiruz Surur, Balkachew Nigatu, Alula Teklu, Tewodros Getinet, Munir Kassa, Merhawi Gebremedhin, Berhe Gebremichael, Yonas Abesha

**Affiliations:** 1 Department of Obstetrics and Gynecology, Saint Paul’s Hospital Millennium Medical College, Addis Ababa, Ethiopia; 2 Research Office, Saint Paul’s Hospital Millennium Medical College, Addis Ababa, Ethiopia; 3 Public Health Department, Saint Paul’s Hospital Millennium Medical College, Addis Ababa, Ethiopia; 4 Federal Ministry of Health, Ministry’s Office, Addis Ababa, Ethiopia; 5 School of Public Health, Haramaya University, Harar, Ethiopia; 6 Private Consultancy Practice, Addis Ababa, Ethiopia

**Keywords:** Contraceptive prevalence rate, Emerging regions, Ethiopia, Reproductive age women

## Abstract

**Background:**

Ethiopia is the second most populous country in Africa, known for its high fertility and low contraceptive use. The magnitude of contraceptive use in the emerging regions of the country is below the national average. However, there is a paucity of evidence regarding the reasons for low contraceptive use in these regions. Therefore, this study aimed to assess contraceptive use and associated factors in the emerging regions of Ethiopia.

**Methods:**

For the quantitative part, a community based cross-sectional study was conducted among 2891 reproductive age women who were selected by multistage sampling technique. Data were collected face to face using an open data kit software, and STATA version 14 was used for data analysis. Frequencies, percentages, summary measures and tables were used to summarize and present the data. Bivariable and multivariable logistic regression analyses were performed to identify factors associated with contraceptive use, by computing odds ratio with 95% confidence interval. Level of significance was considered at *p*-value < 0.05. For the qualitative part, phenomenological study was conducted among 252 health care workers and community members who were selected purposely. The data were collected by focused group discussions, in-depth interviews and key informant interviews. The data were audio-recorded in the local languages, and then translated to English verbatim. NVivo version 11 was used to analyze the data through a thematic analysis method.

**Results:**

The overall contraceptive prevalence rate was 22.2%; with 11.7, 38.6, 25.5 and 8.8% for Afar, Benshangul Gumuz, Gambela and Somali Regions, respectively. Age, religion, education, marital status, family size, ideal children, knowledge and attitude were significantly associated with contraceptive use. Additionally, the qualitative study identified three themes as barriers to contraceptive use: individual, health care system and sociocultural factors.

**Conclusions:**

Contraceptive prevalence rate was low in this study compared to the national average. Age, religion, education, marital status, family size, ideal children, knowledge and attitude were significantly associated with contraceptive use. From the qualitative aspect, individual, health care system and sociocultural factors were identified as barriers to contraceptive use. Therefore, the emerging regions of Ethiopia need special focus in increasing contraceptive use through behavioral influence/change.

## Introduction

Ethiopia is a low income country with poor health service coverage, including family planning (FP), particularly in the emerging regions [1]. The Ethiopian government has made a massive expansion of health facilities and trained human support capacity [2]. Accordingly, contraceptive prevalence rate (CPR) of the country has increased from 8% in 2000 to 41.4% in 2019 [3]. However, there was a significant regional variation in CPR among the emerging regions, with 3% in Somali Region and 38.5% in Benishangul Gumuz (BG) Region. Different factors such as age, education, family size, spousal communication on FP, number of live children, spousal approval on FP, ideal number of desired children, residence, religion, knowledge, and attitudes influence the uptake in contraceptive use [4–7].

Ethiopia has set an ambitious plan to improve primary health care services. To this effect, the Federal Ministry of Health (FMoH) has developed a health sector transformation plan (HSTP), with due emphasis given to FP. Accordingly, CPR was expected to reach 55% by 2020 [8]. However, 41.4% of married women were using modern contraceptive at national level, as of the Ethiopian Mini Demographic and Health Survey of 2019 [3]. Meanwhile, rapid expansion of health facilities/institutions and FP services task shifting to the lower levels, including health posts, has been made progressively since the last decade [2, 9]. Health post and health centers are the front line for FP services delivery and about 16,559 health posts and 3982 health centers are providing contraceptives in the nation [2], granting 90% access to FP [10].

Despite promising changes in the country, both community surveys and facility reports show that contraceptive use in the emerging regions is far below the national goal. The FMoH reported that contraceptive acceptance rate was very low in emerging regions [11]. In contrast, emerging regions are known to be highly polygamous (19 to 29%) in addition to high ideal desire of children (4.5–10.5) [12], with poor access to FP services [13]. To the level of our knowledge, there is no large-scale study done in the emerging regions with depth analysis. In response to this, a large scale study, with quantitative and qualitative approaches, was done to assess the overall contraceptive use and its associated factors among reproductive age women in the four emerging regions of Ethiopia.

## Materials and methods

### Study area and period

The study was conducted in the emerging regions of Ethiopia from 01 to 30 June 2017. According to the Ethiopian government, BG, Gambela, Afar and Somali are considered as emerging regions. Afar and Somali are pastoral regions while BG and Gambela are agrarian regions. According to 2014 population projection, the total population for Afar, BG, Gambela and Somali were 1,678,000 (755,000 females), 976,000 (481,000 females), 396,000 (189,000 females), and 5,307,000 (2,420,000 females), respectively [14].

The health service delivery of Ethiopia has been restructured into a three-tier system. The first tier, the primary health care unit (PHCU), is composed of a health center (HC), satellite health posts (HP), and a primary hospital. While the second and third tiers are general hospitals and specialized hospitals, respectively. Family planning (FP) is provided extensively at the PHCU. According to the current approach, one woreda, with an average population of 100,000, needs to have one primary hospital, four HCs, and 20–33 HPs. So far, about 380 HCs and 1998 HPs are found in the four emerging regions, and the regions are characterized by a low health care provider to population ratio [2].

### Study design and population

A community based cross-sectional study design was applied for the quantitative study while phenomenological study design/approach was used for the qualitative one. For the quantitative data, reproductive age women (15–49 years old) were the study population; whereas health professionals working at different levels and community inhabitants, including community and religious leaders were the study population for the qualitative study. Individuals with serious medical or mental health problems and known infertile women were excluded from the study.

### Sample size determination and sampling procedure

Considering the sociocultural differences among the regions, the sample size was estimated independently for each emerging region. Since there was no similar literature showing the proportion of contraceptive use in the same context, we used *p* = 50% that provides the maximum sample size. Single population proportion formula was used by considering confidence level = 95%, margin of error = 1%, design effect = 2 and 10% for anticipated non-response. Mathematically, ***n***_***h***_ **= (*z***^***2***^**) (*p*) (1-*p*) (*d*) (*r*) / (*c*) (a*)*** **(*e***^***2***^**),** where *n*_*h=*_sample size in terms of number of households to be selected, *z =* level of confidence, *p* = proportion, *d =* design effect, *r =* multiplier to account for the anticipated rate of non-response, *c* = proportion of total population contributed by the target population of the survey, *a =* average household size, and *e*^*2*^ *= margin* of error. Based on the formula, the final sample size was fixed at a total of 2929 women (i.e. 683, 805, 678 and 753 women for Afar, BG, Somali and Gambela Regions, respectively).

Regarding the sampling procedure, all woredas of each emerging region were listed (*woreda is the second smallest administrative unit in Ethiopia*). Accordingly, there were 30, 20, 53 and 13 woredas in Afar, BG, Somali and Gambela Regions, respectively. In the first stage, 20% of the total woredas were selected from each region by lottery method (i.e. 6, 4, 10 and 3 woredas for Afar, BG, Somali and Gambela Regions, respectively). Then, five kebeles (*kebele is the smallest administrative unit in Ethiopia*) were randomly selected from each woreda. In the third stage, two enumeration areas were randomly selected from each kebele. One enumeration area is expected to have an average of 80 households. Fresh households which were not documented in the lists received from woreda/kebele files were recorded by the research team and included in the sampling process. Finally, a systematic random sampling was used to select the actual participants of the study (i.e. the reproductive age women). Sampling intervals of 7, 4, 12 and 3 were calculated for Afar, BG, Somali and Gambela Regions, respectively, by dividing the total household list to the expected sample per enumeration areas. Lottery method was used to select the first household from the interval for each region. In case a household has two or more eligible women, one of them was selected using lottery method for interview.

For the qualitative part, a total of 252 individuals participated who were selected purposely; it included 15 focused group discussions (FGDs) (*n* = 120), 80 in-depth interviews (IDIs) (*n* = 80) and 52 key informant interviews (KIIs) (*n* = 52). Four FGDs were conducted in each region, except for Afar Region which were three FGDs. In addition, 20 IDIs and 13 KIIs were conducted in each region. The study participants for the FGDs were selected separately for male and female groups. Accordingly, eight of the FGDs were female groups and the remaining seven were male groups. The participants in the FGDs were community members, including community (clan) and religious leaders. The number of participants per FGD ranges from 6 to 12 individuals, and all discussions were conducted for duration of one to 2 h. On the other hand, 20 IDIs and 13 KIIs were conducted in each region. The participants for the IDIs were reproductive age women, and the participants for the KIIs were health care workers, and regional and woreda FP services coordinators. Each interview took duration of 30 min to an hour.

### Data collection methods

For the quantitative study, a questionnaire was adapted from different prior literature and formatted to best respond to research questions. The questionnaire includes questions on socio-demography, reproductive history, and FP knowledge, attitude and utilization. The questions were developed and constructed by the investigators after reviewing different previous literature. Repeated discussions were conducted among the investigators during the development of the questionnaire. Each question was critically assessed in these group discussions to align with the research questions and objectives. Then, first draft of the questionnaire was reviewed by the FMoH technical team of expert, and their feedback was incorporated. The questionnaire was initially prepared in English, and was later translated into three local languages (Amharic, Somali, and Afar languages) by language experts for the purpose of data collection.

Data collectors and supervisors, who were native local languages speakers and health related professionals, were recruited and deployed to the data collection process. A survey operation guideline (SOG) was developed, and four-day training was provided by the investigators to assist field staffs. Most of the data collectors and supervisors were recruited from the respective regions; the training was conducted in each region separately. Data were collected face to face by the mobile application, open data kit (ODK). The overall data collection was supervised by the investigators centrally. The investigators had routine communications with the data collectors, supervisors and data manager through field visits, phone calls and social media communications. Supervisors were closely following and supporting the data collectors at the field sites and were communicating with the investigators regarding the data collection process on daily basis. Since the data were collected using ODK, the data collectors submit their data to central server and communicate with the investigators, supervisors, and data manager for any support they want during the field work. If connection was absent, the data collectors were able to save the data and continue data collection after reconnecting to an internet source. The data were submitted to and stored in a central data storage system (server) and the data manager at the central office routinely monitored the incoming data, notifying field staff and investigators if any potential errors occurred.

For the qualitative part, the study used FGD, IDI and KII guides to collect the data. The guiding questions were developed and assessed critically by the investigators and FMoH technical team of experts to fit with the research question (i.e. to find out the barriers of contraceptive use). The tool was initially developed in English and translated into the local languages by experts. Then, it was pretested before the actual data collection. Data collectors and supervisors were recruited considering fluency in the local language and previous experience in qualitative data collection/supervision. Four-day training was provided both to the data collectors and supervisors regarding the data collection process. The data collection team for the FGDs had male and female interviewers and collected the data from the male and female groups separately until the saturation of ideas in at least two consecutive FGDs. The collected data from the aforementioned three sources (i.e. from FGDs, IDIs and KIIs) were audio-recorded.

### Operational definition

***Emerging regions:*** In Ethiopia, emerging regions are defined as least developed regions characterized by poor level of infrastructure and services; border and internal (clan) conflicts; strongly traditional in social practices; predominantly rural with small, scattered and nomadic population; dominated by pastoral and agro-pastoral as the basic means of livelihood; gender inequalities; poor capacity of regional and local government systems to deliver basic infrastructures and services [15].

***Contraceptive prevalence rate (CPR):*** is the proportion of reproductive age women who were using at least one modern contraceptive method during the study period; i.e. the proportion of current contraceptive users.

***Contraceptive use:*** is the utilization of at least one modern contraceptive method by reproductive age women.

***Current contraceptive users:*** are reproductive age women who were using at least one modern contraceptive method during the study period.

### Data quality control

The questionnaire was translated into the local languages; i.e. Afar, Amharic and Somali languages for data collection and then translated back into English for analysis and reporting. The questionnaire was reviewed and assessed by technical team of experts from FMoH. Before the actual data collection, 5% of the questionnaires were pretested in woredas which were not included in this study. Data collectors and supervisors were trained for 4 days on the data collection process to have a common understanding. The data collectors were closely supervised by the supervisors and investigators. Completeness of each questionnaire was checked by the investigators and data manager on daily basis. To minimize transcription and translation inconsistencies of the qualitative data, the transcribed and translated data were compared to the audio-recorded one by other individuals with similar background and educational level.

### Data processing and analysis

The quantitative data were first downloaded from the server as an Excel (.xls) files, and then exported to STATA version 14. Data were checked for completeness, coded, and cleaned before analysis. The characteristics of study participants was described and presented using frequencies, percentages, and summary measures such as mean and standard deviation (SD).

To assess knowledge of the participants, 12 questions related to the types of contraceptive methods were asked. Each question’s response was coded as “1” for “yes” and “0” for “no”. The internal consistency (α) of the items was checked and found to be 0.87, which indicates an acceptable level of reliability. Then, score was computed for each participant, which ranges from 0 to 12. The score was tested for normality and was found normally distributed. Finally, a composite knowledge variable was created from the score considering mean as a cut-off-point. Participants with a score of mean and above were classified as having “good knowledge”, and those with a score of below the mean were categorized as having “poor knowledge”.

Similarly, attitude of the participants on contraceptive methods was assessed using 9 attitude related questions. Some questions were in the form of a Likert scale while others were not. Thus, all the questions were re-coded in binary form. The internal consistency (α) of the items was found to be 0.78, indicating acceptable reliability. Just as for the knowledge assessment, attitude score was computed for each participant, ranging from 0 to 9. The score was tested for normality and was normally distributed. Finally, a composite variable was created from the score considering mean as a cut-off-point. Participants with a score of mean and above were classified as having a “favorable attitude” whereas those with a score of below the mean were categorized as having an “unfavorable attitude”.

Contraceptive use (the dependent variable) was coded as “0” for non-users and “1” for users. A bivariable logistic regression analysis was performed to assess the association between the dependent variable and each independent variable. Variables with *p*-value < 0.25 from the bivariable logistic regression analysis had been included in the final multivariable logistic regression analysis model to control for confounding factors. Multicollinearity test was done among the independent variables using variance inflation factor (VIF), and no significant (VIF > 10) collinearity was detected. A model goodness-of-fit was checked by a Hosmer-Lemeshow test and the model was well fitted (*p* = 0.43).

The collected qualitative data were first transcribed in the local languages and translated to English by native speakers of the local languages who had BSc degree in the field of health sciences. Then, the data were exported into NVivo version 11 for analysis and coding was done line by line, segment by segment, and paragraph based. All coded items were categorized into specific themes and sub-themes. Thematic analysis was applied to derive the main themes and sub-themes, and the results are presented by narrative approach.

## Results

### Socio-demographic characteristics of participants

Twenty-three woredas (Somali = 9, Afar = 6, BG =4, and Gambela = 4) were included in this study. With 99.1% overall response rate, 643 (22.2%), 794 (27.5%), 752 (26%), and 702 (24.3%) of participants were from Afar, BG, Gambela, and Somali Regions, respectively. Mean age of the participants was 26.8 (± 7.5 SD) years, 2135 (73.8%) were from rural area, and 1525 (52.7%) had no formal education. More than three-fourth (76.5%) of participants were married or in union and 1713 (59.3%) were Muslims. The overall mean family size of the participants’ households was 5.9 persons (+ 2.8 SD), with the highest mean family size in Somali Region (6.7 + 3.2 SD) followed by Afar and Gambela Regions (6.1 + 2.7 SD for each). Regarding occupation, 89.4, 75.9, 58.2, and 75.1% of participants from Afar, BG, Gambela and Somali Regions were daily laborers/farmers, respectively. There was no participant from Afar Region with a monthly household income greater than the median (1000 Ethiopian Birr (ETB)), but participants from BG (55.5%), Gambela (48.8%) and Somali (67.6%) Regions reported monthly income greater than the median (1000 ETB). Six hundred twenty-six (21.6%) of participants had radio, 544 (18.8%) had television, and 1373 (47.5%) had their own cellphone. One-fourth (26.4%) of participants had history of watching/listening/reading TV/radio/newspaper at least once a week (Table 1).

**Table 1:** Socio-demographic and reproductive characteristics of reproductive age women in four emerging regions of Ethiopia (in frequencies and percentages), 2017

Background characteristics	Emerging regions				
	Afar (***n*** = 643)	BG (***n*** = 794)	Gambela (***n*** = 752)	Somali (***n*** = 702)	Total (***n*** = 2891)
Rural residence	468(72.8)	652(82.1)	528(70.2)	487(69.4)	2135(73.8)
Age (in years)					
15–24	222(34.5)	326(41.0)	383(51.0)	260(37.0)	1191(41.2)
25–34	331(51.5)	292(36.8)	271(36.0)	305(43.5)	1199(41.5)
35–49	90(14.0)	176(22.2)	98(13.0)	137(19.5)	501(17.3)
Educational status					
No education	504(78.4)	305(38.4)	219(29.1)	497(70.8)	1525(52.7)
Primary	107(16.6)	318(40.1)	279(37.1)	95(13.5)	799(27.6)
Secondary	17(2.6)	93(11.7)	178(23.7)	72(10.3)	360(12.5)
Above Secondary	15(2.3)	78(9.8)	76(10.1)	38(5.4)	207(7.2)
Partner’s education					
No education	414(64.4)	207(26.1)	170(22.6)	359(51.1)	1150(39.8)
Primary	77(12)	238(30)	113(15)	36(5.1)	464(16.1)
Secondary	27(4.2)	97(12.2)	125(16.6)	57(8.1)	306(10.6)
Above Secondary	28(4.4)	94(11.8)	181(24.1)	53(7.5)	356(12.3)
Not Applicable	97(15.1)	158(19.9)	163(21.7)	197(28.1)	615(21.3)
Family monthly income > median(1000 ETB)	0	441(55.5)	366(48.8)	474(67.6)	1281(44.4)
Muslim in religion	622(96.7)	387(48.7)	26(3.5)	678(96.6)	1713(59.3)
Employment status					
Labor work	575(89.4)	603 (75.9)	438 (58)	527(57)	2143(74.1)
Merchant	22(3.4)	50(6.3)	35(4.6)	68(9.7)	175 (6.1)
Employee	21(3.2)	48(6)	74(9.8)	34(4.8)	177(6.12)
Student	25(3.8)	93(11.7)	205(27.3)	73(10.4)	396 (13.7)
Having radio/TV/mobile phone	386(60)	482(60.7)	390(51.8)	432(61.5)	1690(58.5)
Listening Radio/TV at least once a week	183(28.5)	309(38.9)	109(14.5)	161(23)	762(26.4)
Family size					
1–5	292(45.4)	504(63.5)	353(46.9)	271(38.6)	1420(49.1)
6–10	314(48.8)	275(34.6)	358(47.6)	352(50.1)	1299(44.9)
> =11	37(5.8)	15(2)	41(5.4)	79(11.3)	172(6)
Ideal number of children > mean (5 children)	(536(83.4)	401 (50.5)	573(76.2)	666(94.9	2176(75.3)
Ever had sexual intercourse	590(91.7)	708(89.2)	603(80.2)	573(81.6)	2474(85.6)
Mean Age at first sex	16.6(2.3)	16.8(3.1)	17.2(2.8)	18(2.9)	17.2(2.8)
Practice sex before 18 years	363(71.5)	411(61)	368(60.6)	244(44.2)	1386(59.2)
Last pregnancy wanted	353(68.7)	498(78.5)	320(59)	501(96)	1672(75.6)
Total number of pregnancy > = mean (5 children)	233(45.3)	212(33.4)	158(29.2)	281(53.8)	884(40)
Ever face abortion	54(8.4)	110(13.9)	57(7.6)	75(10.7)	296(13.4)
Ever face stillbirth	0	50(7.9)	38(7.1)	32(6.1)	120(5.4)
Having live children > 4	136(26.5)	130(20.5)	75(13.8)	164(31.4)	505(22.8)

### Reproductive characteristics of participants

Self-reported numbers of women who had ever had sexual intercourse was 85.6% (91.7,89.2, 80.2 and 81.6% for Afar, BG, Gambela, Somali Regions, respectively). Mean age of participants at first sex was 17.2 + 2.8 SD years. Of those, who ever had sex, 71.5, 61, 60.6 and 44.2% participants from Afar, BG, Gambela and Somali, respectively, had sex before turning 18 years old. More than three-fourth (76.5%) had history of pregnancy where the last pregnancies were unwanted for 24.4% of participants. Mean age at first pregnancy was 18.5 (+ 2.9 SD) years and average number of live children was 4.2 + (2.7 SD); average live children was higher for Somali women (4.4 + 2.8 SD) while lower for BG (3 + 2.1 SD). More than one-third (69.3%) of the participants had a desire to have additional children in the future. Mean desire of ideal children was 7.5 (+ 3.5 SD) with the highest mean ideal children (10.2 + 3.5SD) in Somali Region and lowest in BG Region (5.5 + 2.9 SD). About 5.4 and 13.4% of ever pregnant mothers reported a history of stillbirth and abortion, respectively (Table 1).

### Knowledge and attitude of women on family planning

The study showed that 2394 (82.8%) of the participants had knowledge of at least one contraceptive method. Only one third (33%) knew six or more (half of the list) contraceptives types. The mean knowledge score of FP was 4.3 (+ 3.4 SD) and 1254 (43.4%) of the respondents had good FP knowledge. Good knowledge was higher for BG (62.7%) followed by Gambela (41.1%) women. The most common contraceptive methods among participants were injectables, (72.3%), followed by pills (67.6%), and implants (51.7%). Health Extension Workers (HEWs) were the major source of contraceptive methods for 45.2% of the respondents, with health care providers (28.8%) and friends (24.7%) following as sources for the participants (Table 2).

**Table 2:** Knowledge of contraceptive methods (in frequencies and percentages) among reproductive-age women in the four emerging regions of Ethiopia, 2017

Variable	Emerging regions				
	Afar (***n*** = 643)	BG (***n*** = 794)	Gambella (***n*** = 752)	Somali (***n*** = 702)	Total (***n*** = 2891)
Know at least one contraceptive method	581(90.4)	761(95.8)	538(71.5)	514(73.2)	2394(82.8)
Know male condom	313(48.7)	475(59.8)	397(52.8)	200(28.5)	1385(47.9)
Know pill	462(71.9)	664(83.6)	428(56.9)	400(57.0)	1954(67.6)
Know injectable	506(78.7)	730(91.9)	446(59.3)	407(58.0)	2089(72.3)
Know IUD	95(14.8)	471(59.3)	153(20.3)	96(13.7)	815(28.2)
Know implants	211(32.8)	696(87.7)	336(44.7)	252(35.9)	1495(51.7)
Know tubal ligation	73(11.4)	204(25.7)	132(17.6)	47(6.7)	456(15.8)
Know vasectomy	56(8.7)	97(12.2)	115(15.3)	38(5.4)	306(10.6)
Female condom	77(12.0)	173(21.8)	256(34.0)	71(10.1)	577(20.0)
Know LAM	349(54.3)	244(30.7)	158(21.0)	368(52.4)	1119(38.7)
Know rhythm	230(35.8)	388(48.9)	285(37.9)	224(31.9)	1127(39.0)
Know withdrawal	109(17.0)	171(21.5)	72(9.6)	76(10.8)	428(14.8)
Know emergency contraceptive	141(21.9)	187(23.6)	181(24.1)	122(17.4)	631(21.8)
Good overall knowledge (composite)	222(34.5)	498(62.7)	309(41.1)	225(32.1)	1254(43.4)
Source of contraceptive information					
Friends	170(26)	208(26.2)	174(23.1)	163(23.2)	715(24.7)
School	90(14)	186(23.4)	158(21)	77(11)	511(17.7)
Family	73(11)	95(12.0)	44(5.9)	47(6.7)	259(9)
HEWs	310(48.2)	561(70.7)	276(36.7)	159(22.6)	1306(45.2)
Health care providers	220(34.2)	300(37.8)	124(16.5)	190(27.1)	834(28.8)
Radio	107(16.6)	93(11.7)	4(0.5)	25(3.6)	229(7.9)
Television	62(9.6)	92(11.6)	40(5.3)	63(9)	257(8.9)
Printed-materials	8(1.2)	3(0.4)	0(0.0)	1(0.1)	12(0.4)
Internet/Social Media	8(12)	1(0.1)	4(0.5)	20(2.8)	33(1.1)

The mean score for the overall attitude of FP was 4.0 (+ 2.5 SD), and attitude mean score was higher among current contraceptive users (6.1 + 1.8 SD) compared with non-users (4 + 2.4 SD). More than half (52.3%) of the respondents had a favorable attitude (> mean score) towards FP. The mean attitude score of contraceptive use was also differed by location of residence where 4.5 (+ 2.4 SD) for urban residents and 3.8 (+ 2.5 SD) among rural dwellers. Meanwhile, there was a high regional variation in attitude mean score of contraceptive use with the lowest score in Somali Region (2.4 + 1.8 SD) and highest score in BG Region (5.8 + 2SD) (Table 3).

**Table 3:** Attitude of contraceptive methods (in frequency and percentage) among reproductive-age women in the four emerging regions of Ethiopia, 2017

Variable	Emerging regions				
	Afar (***n*** = 643)	BG (***n*** = 794)	Gambella (***n*** = 752)	Somali (***n*** = 702)	Total (***n*** = 2891)
Discussion with one’s partner on FP					
Frequently	29(6.1)	177(28.0)	115(24.1)	35(7.1)	356(17.1)
Sometimes	51(10.7)	239(37.8)	109(22.8)	30(6.0)	429(20.6)
Rarely	78(16.3)	58(9.2)	20(4.2)	36(7.3)	192(9.2)
Never	320(66.9)	159(25.1)	234(49.0)	395(79.6)	1108(53.1)
Discussion with family/relative					
Frequently	14(2.2)	52(6.5)	25(3.3)	6(0.9)	97(3.4)
Sometimes	53(8.2)	220(27.7)	108(14.4)	29(4.1)	410(14.2)
Rarely	68(10.6)	116(14.6)	64(8.5)	70(10.0)	318(11.0)
Never	508(79.0)	406(51.1)	555(73.8)	597 (85.0)	2066(71.5)
Discussion with friends					
Frequently	23(3.6)	66(8.3)	51(6.8)	14(2.0)	154(5.3)
Sometimes	67(10.4)	239(30.1)	180(23.9)	53(7.5)	539(18.6)
Rarely	77(12)	143(18.0)	79(10.5)	73(10.4)	372(12.9)
Never	476(74.0)	346(43.6)	442(58.8)	562(80.1)	1826(63.2)
Contraception has benefit for women					
Yes	264(41.1)	691(87.0)	452(60.1)	323(46.0)	1730(59.8)
No	194(30.2)	45(5.7)	95(12.6)	110(15.70)	444(15.4)
Don’t know	185(28.8)	58(7.3)	205(273)	269(38.3)	717(24.8)
Large family size affects economic condition negatively					
Strongly agree	54(8.4)	257(32.4)	163(21.7)	37 (5.3)	511(17.7)
Agree	174(27.1)	417(52.5)	259(34.4)	123(17.5)	973(33.7)
Neutral	52(8.1)	31(3.9)	91(12.1)	51(7.3)	225(7.8)
Disagree	269(41.8)	69(8.7)	178(23.7)	349(49.7)	865(29.9)
Strongly disagree	94(14.6)	20(2.5)	61(8.1)	142(20.2)	317(11.0)
Large family size earns respect by the husband					
Strongly agree	129(20.1)	32(4.0)	78(10.4)	138(19.7)	377(13.0)
Agree	226(35.1)	237(29.8)	144(19.1)	324(46.2)	931(32.2)
Neutral	69(10.7)	77(9.7)	99(13.2)	79(11.3)	324(11.2)
Disagree	182(28.3)	419(52.8)	325(43.2)	119(17.0)	1045(36.1)
Strongly disagree	37(5.8)	29(3.7)	106(14.1)	42(6.0)	214(7.4)
Large family size earns the respect of the community					
Strongly agree	107(16.6)	26(3.3)	75(10.0)	157(22.4)	365(12.6)
Agree	212(33.0)	252(31.7)	164(21.8)	327(46.6)	955(33.0)
Neutral	92(14.3)	87(11.0)	111(14.8)	81(11.5)	371(12.8)
Disagree	183(28.5)	406(51.1)	294(39.1)	105(15.0)	988(34.2)
Strongly disagree	49(7.6)	23(2.9)	108(14.4)	32(4.6)	212(7.3)
Large family size earns respect by a religious leader					
Strongly agree	156(24.3)	18(2.3)	98(13.0)	122(17.4)	394(13.6)
Agree	169(26.3)	253(31.9)	163(21.7)	297(42.3)	882(30.5)
Neutral	120(18.7)	171(21.5)	101(13.4)	122(17.4)	514(17.8)
Disagree	152(23.6)	329(41.4)	248(33.0)	135(19.2)	864(29.9)
Strongly disagree	46(7.2)	23(2.9)	142(18.9)	26(3.7)	237(8.2)
Large family size affects maternal and child health					
Strongly agree	78(12.1)	190(23.9)	125(16.6)	46(6.6)	439(15.2)
Agree	168(26.1)	455(57.3)	263(35.0)	266(37.9)	1152(39.8)
Neutral	79(12.3)	44(5.5)	106(14.1)	88(12.5)	317(11.0)
Disagree	250(38.9)	94(11.8)	198(26.3)	240(34.2)	782(27.0)
Strongly disagree	68(10.6)	11(1.4)	60(8.0)	62(8.8)	201(7.0)
Overall attitude (composite)					
Favorable	214(33.3)	687(86.5)	464(61.7)	146(20.8)	1511(52.3)
Unfavorable	429(66.7)	107(13.5)	288(38.3)	556(79.2)	1380(47.7)

### Contraceptive prevalence rate

The overall CPR was 579 (22.2%); it was 11.7, 38.6, 25.5, and 8.8% for Afar, BG, Gambela and Somali Regions, respectively. The contraceptive prevalence rate (CPR) was higher among urban than rural women (28.4% versus 19.9%). Similarly, CPR was higher among Christian than Muslim women (32.3% versus 15%). The contraceptive prevalence rate (CPR) was 12.4, 32.2, 29 and 43.3% for women with no formal education, primary level, secondary level, and above secondary level, respectively. Contraceptive prevalence rate (CPR) was higher among employed than daily laborer/farmer women (41% versus 21.6%). Women with family monthly income above the median (26.8%) had higher CPR than those with less than the median income (18.5%). Besides, women who listen/watch radio/TV at least once per week had higher CPR (34%) compared with those who never listen/watch (18.1%). Women with good knowledge and favorable attitude had CPR of 36.1 and 38.2%, respectively.

### Factors associated with contraceptive use

The bivariable logistic regression analysis result showed that large family size, having high number of live children, and desiring high number of ideal children were negatively associated with contraceptive use. Similarly, being from urban area, older age, being married or in union, being Christian, higher education level, having an educated husband, having radio/TV, listening/watching radio/TV, high monthly family income, being employed, and having good knowledge and favorable attitude towards FP were positively associated with contraceptive use (*p* < 0.05) (Table 4).

**Table 4:** Factors associated with current contraceptive use among reproductive age women in four emerging regions of Ethiopia, 2017

Independent variables	Categories	Contraceptive use		COR (95% CI)	AOR (95% CI)
		Yes	No		
Residence	Rural	382	1531	1.00	1.00
	Urban	197	496	1.6(1.3, 1.9)**	1.1 (0.8, 1.6)
Age	15–24	223	658	1.00	1.00
	25–34	234	613	1.1(0.9, 1.3)	1.2 (0.9, 1.8)
	35–49	62	333	0.5(0.4, 0.7) *	1.6(1.1, 2.4)*
Marital status	Single	39	478	1.00	1.00
	Married	519	1423	4.5(3.2, 6.2)**	8.4(5.5, 12.7)**
	Others	21	126	2(1.1, 3.6)*	4.5(2.0, 9.9)**
Family size	1–5	407	874	1.00	1.00
	6–10	167	999	0.4(0.3, 0.4)**	0.65(0.5, 0.8)*
	> 11	5	154	0.1(0.03, 0.2)**	0.2(0.1, 0.5)*
Religion	Muslim	228	1290	1.00	1.00
	Non-Muslim	351	737	2.7(2.2, 3.3)**	1.3(1.1, 1.7)*
Level of education	No	168	1192	1.00	1.00
	Primary	235	496	3.4(2.6, 4.2)**	2.1(1.6, 2.8)**
	Secondary	95	233	2.8(2.1, 3.8)**	1.8(1.2, 2.7)*
	Above secondary	81	106	5.4(3.8, 7.5)**	2.1(1.3, 3.4)*
Husband level of education	No	121	891	1.00	1.00
	Primary	165	246	4.9(3.7, 6.4)**	1.4(1.0, 2.1
	Secondary	111	154	5.3(3.8, 7.2)**	1.1(0.7, 1.8)
	Above secondary	126	183	5.1(3.7, 6.8)**	1.1(0.6, 1.9)
Occupation	Farmer	410	1492	1.00	1.00
	Merchant	50	118	1.5(1.1, 2.1)*	1.2(0.7, 2.4)
	Employee	66	95	2.5(1.8, 3.5)**	1.2(0.6, 2.4)
	Student	53	322	0.59(0.4, 0.8)**	1.2(0.60, 2.2)
Household monthly income	< 1000 ETB	266	1175	1.00	1.00
	> 1000 ETB	312	850	1.6(1.3, 1.9)**	1.1(0.8, 1.5)
Having TV/Radio	No	419	1688	1.00	1.00
	Yes	160	339	1.9(1.5, 2.4)**	1.1(0.8, 1.6)
Watch/listening TV/radio	No	344	1543	1.00	1.00
	Yes	235	484	2.2(1.8, 2.6)**	1.1(.8, 1.6)
Total number of live children	< 4	506	1491	1.00	1.00
	> 4	73	536	0.42(0.3, 0.5)**	0.8(0.5, 1.2)
Number of ideal desired children	1–4	260	394	1.00	1.00
	> 5	319	1633	0.3(0.2, 0.4)**	0.8(0.6, 0.9)*
Knowledge on FP	Poor	169	1302	1.00	1.00
	Good	410	725	4.4(3.0, 5.3)**	6.6(4.6, 9.6)**
Attitude towards FP	Unfavorable	54	1176	1.00	1.00
	Favorable	525	851	13.4(10.0, 18.0)**	2.1(1.6, 2.8)**

* = *p*-value < 0.05, ** = *p*-value< 0.001, *CI* Confidence interval, *COR* Crude odds ratio, *AOR* Adjusted odds ratio

In the multivariable logistic regression analysis, being in the age group of 35–49 years, being married, being Christian, primary level or more education, and having good knowledge and favorable attitude towards FP were positive predictors of contraceptive use. Whereas, having large family size and high ideal children were negatively associated with contraceptive use (*p* < 0.05) (Table 4).

In addition to the quantitative result, three themes of barriers of contraceptive use were identified from the qualitative study. These are individual, health care system and sociocultural factors.

The individual factors include four sub-themes: poor FP knowledge, lack of women’s decision-making power, fear of side effects, and being busy. Poor FP knowledge was reported by most of the participants as one of the primary bottlenecks to contraceptive use. The participants also responded that fear of side effects was a barrier to use contraceptive methods in the study areas. Moreover, most of the participants agreed that contraceptive use was primarily decided by husbands and this was higher for rural than urban residents. Participants also indicated that there are women who were unable to properly use contraceptive methods due to being busy of domestic workloads.

*“.....we want to use contraceptive methods, but we fear the side effects such as excessive bleeding and infertility. In addition, men are decision makers and have great role in protecting us from using contraceptives. My husband decides on me regarding every activity done in our life. There are women in our community who have interest to use contraceptives, but refused by their husbands.”* ***IDI participant (reproductive age woman), from Afar Region***

The health care system factors include four sub-themes: lack of accessibility to contraceptive methods, low financial support, lack of guiding policy, and low monitoring system. Majority of the respondents concluded that poor transportation access, lack of roads, long distances to access health facilities and shortage of available contraceptive methods were the reasons to low contraceptive use. Shortage of funds at the health facility and health administration levels was also indicated as a major reason for low contraceptive use. Health professionals and FP service coordinators indicated that even though they had good plans and strategies regarding FP services, they were unable to implement them due to budget problems. In addition, they pointed out that there were poor monitoring systems and guiding policies on FP for the emerging regions.

*“ … because of distance and poor road infrastructures, it is difficult to provide FP services; the areas are not accessible to transport, especially during the summer. There are also problems that affect the provision of FP services such as shortage of budget, poor monitoring systems and lack of clear policies and guidelines.”* ***KII participant, FP service coordinator from Somali Region***

The sociocultural factors include seven sub-themes: male dominance, desire for large family size, poor attitudes towards FP, a nomadic lifestyle, myths on FP, fear of adultery, and poor FP acceptance. Almost all participants agreed that a woman who wishes to use contraceptive methods should first obtain her husband’s approval. Women who use contraceptives without the knowledge of their husbands may face divorce, physical attack, family conflict, polygamy and mistrust. Another factor which was reported as a reason to low contraceptive use was the desirability of a large family. Communities in these emerging regions generally want to have a large family size in order to cope up with inter and intra clan competitions. As a result, clan leaders do not allow their clan members to use contraceptive methods in order to increase clan size.

Participants also reported that there is a belief in the community that modern contraceptive use requires a balanced diet and failure to do so results in negative health outcomes. Thus, women with shortage of food are not recommended taking contraceptives. Another reported misperception was some individuals believe the provision of FP is carried out in order to accomplish government interests, and the community is not yet well convinced on the issues of FP. Some individuals also believe that contraceptive use is perceived as killing the already conceived life and therefore is considered as a sin by the followers of some religions.

*“ … we need to have children with no gaps and nothing used. Allah feeds for all, including our children. Our community should not use contraceptives. We are Muslims; we do not use contraceptives because our religion does not allow us to do so and utilization is considered as sin.”* ***Female FGD participant, from Somali Region***

Another finding from this report indicated that letting a wife use contraceptives is equivalent to letting her commit adultery. There is a belief in the community that if a mother uses contraceptives, she will have large periods of time between births, making her healthy, beautiful, and very attractive to other men. Consequently, she may commit adultery.

*“.....when a woman starts to use contraceptives, she has more time to care herself well and becomes beautiful and attractive. This can lead her to commit adultery which may finally result in divorce and other social problems. Therefore, I do not recommend women to use contraceptive methods.”* ***Male FGD participant, from BG Region***

## Discussion

With the objective to assess the magnitude of current contraceptive use and associated factors, this study revealed that the overall CPR was 22.2%; high variation was observed in CPR among regions (11.7% in Afar, 38.6% in BG, 25.5% in Gambela and 8.8% in Somali). Age, marital status, family size, religion, educational status, ideal desire of children, knowledge and attitude were found statistically significant predictors for current contraceptive use. Additionally, the qualitative finding identified three themes (individual, health care system and sociocultural related factors) as main barriers of contraceptive use.

The overall CPR in this study was lower than study findings from different parts of Ethiopia, which ranges between 38.3% in Mojo (Oromia Region) and 82.6% in Bench Maji (Southern Nations, Nationalities and Peoples Region) [5, 8, 16–20]. Moreover, the prevalence was lower than study reports from Turkey (53%) [21] and Haryana state of India (62%) [22]. These variations may be attributed to the differences in culture, geographic location, religious perspectives, and scope of the study including study setting. Besides, women living in emerging regions have lower access to contraceptive methods and contraceptive acceptance rate [11]. Furthermore, the qualitative aspect of this study identified individual, health care system and sociocultural barriers against contraceptive use, and these barriers might have contributed to the low CPR in this study.

Inter-regional variation was also observed in CPR. The current study showed CPR was 11.7% for Afar Region which is almost similar with the finding of the Ethiopian Mini Demographic and Health Survey of 2019 (12.7%) [3], but higher than another study done in the region (8.5%) [23]. The current CPR of BG Region (38.6%) was similar with the aforementioned national mini survey finding (38.5%), while the CPR of Somali Region (8.8%) was a bit higher than the national mini survey finding (3%). In Gambela Region, CPR was 25.5% which is lower than the national mini survey finding (33.8%). These all discrepancies may be attributed to differences in sample size and scale of the studies.

In this study, urban and rural residence was not stratified; rather it was randomly selected, potentially giving over representation of urban residents which is ideally assumed as high contraceptive users. Pocket study done in the Kebri Beyah of Somali Region showed similar finding with the current study [10]; however, studies done in the Assosa Woreda of BG Region showed significant variation with the current findings [24]. This variation may be related with sample size and residence of study participants.

The participants in the age group of 35–49 years were more likely to use contraceptives compared to those in the age group of 15–24 years. This indicates that women mostly start using contraceptives after they achieve the desired number of children. Women also get more freedom from husband pressure as they get older, hence this may give them an opportunity to use more contraceptives than younger women. This finding was in line with studies done in Dodota Woreda (Oromoia Region of Ethiopia) [7], but contradicts with study done in the town of Arba Minch (Southern Nations, Nationalities and Peoples Region, Ethiopia) [8].

Religion was also found to be an important predictor of contraceptive use that Christians were more likely to use contraceptives compared to Muslims. As insured by the qualitative study, the Muslim religion encourages its followers to have more children, and therefore may see using contraceptives as against this goal.

Married women were more likely to use contraceptives than unmarried ones. Similarly, family size categories of 6–10 and 11 or above were at lower odds of contraceptive use than those with family size of 1–5. Meanwhile, desiring an ideal number of children greater than five was negatively associated with current contraceptive use. This finding contradicts with a research conducted in the town of Modjo, Ethiopia [18], where higher fertility was a significant predictor of contraceptive use; this difference may be attributed to sample size, scope of the study, and sociocultural character of participants. Logically, those with family size less than five are younger than their respective counter parts; as a result, the young could be more educated than the older groups as educational resources are improving with time in developing countries. Moreover, young generations use social media more; hence, they may have better access to FP than older generation. Older generations could also stick highly towards religion and culture. It is also logical that women who desire minimum ideal children can use contraceptives to fulfill their objectives. The impact of different socio-demographic variables like a high level of education, high income, and residence, or low desire of ideal children can indirectly affect the use of contraceptives.

Educational level of participants was identified as a significant factor of contraceptive use in this study. Women with education levels of primary, secondary and above secondary were more likely to use contraceptives than less educated women. Educated women can better understand the advantage of FP use. Women’s power of decision-making and personal income also increases with education level which ultimately helps couples to accept contraceptive use easier. Different studies conducted in different part of Ethiopia and other countries have revealed similar finding with this study [7, 8, 17, 18, 23, 25, 26].

Participants who had a good knowledge and favorable attitude on FP were more likely to use contraceptive than their respective counterparts. Even though education and knowledge have not shown any co-linearity, they are interrelated to each other in that mothers may have favorable attitude as a result of education and good knowledge of FP. Having good knowledge is a prerequisite to having a favorable attitude–both good knowledge and favorable attitude are prerequisite to be a FP user provided that an informed choice is an option. This finding was in line with pocket studies conducted in Ethiopia and elsewhere in other countries [4, 8, 20, 23, 26–28]. It is also supported by the qualitative results of this study that poor knowledge, poor attitude and poor acceptance of FP were the barriers to contraceptive use.

### Strength and limitations of the study

The strength of the study is it used large sample size, is multi-center (involving four regions) and covered quantitative and qualitative approaches. However, the study might not show the causal relationship since it used cross-sectional design.

## Conclusions

Overall, CPR was low (22.2%) in the emerging regions of Ethiopia compared to the national magnitude, which is 41.4%. Age, marital status, family size, religion, education, ideal number of children, and knowledge and attitude toward FP were important predictors for current contraceptive use among women in the emerging regions. High regional variation in CPR was observed. Most of the factors that affect the contraceptive use can be improved through behavioral change. Therefore, FMoH, the respective regional health bureaus, and developmental partners should work on improving mass communication through media outlets, and health education by health extension workers should be further strengthened. Similarly, woreda health offices along with community representatives, religious leaders, and local stakeholders should give more emphasis on disseminating FP related messages; this would ultimately bring positive change through improved FP usage.

## Data Availability

Data will be made available from corresponding author upon requesting.

## Abbreviations

AOR: Adjusted odds ratio
BG: Benshangul gumuz
CI: Confidence interval
COR: Crude odds ratio
CPR: Contraceptive prevalence rate
ETB: Ethiopian birr
FGD: Focused group discussion
FMoH: Federal ministry of health
FP: Family planning
HC: Health center
HP: Health post
IDI: In-depth interview
KII: Key informant interview
ODK: Open data kit
PHCU: Primary health care unit
SD: Standard deviation
SOG: Survey operation guideline
VIF: Variance inflation factor
